# Pharmacotherapy considerations with finerenone in the treatment of chronic kidney disease associated with type 2 diabetes

**DOI:** 10.1093/ajhp/zxad192

**Published:** 2023-08-26

**Authors:** Emily Ashjian, Megan Clarke, Kristen Pogue

**Affiliations:** Pharmacy Innovations & Partnerships, Michigan Medicine, Ann Arbor, MI; Department of Clinical Pharmacy, University of Michigan College of Pharmacy, Ann Arbor, MI, USA; Department of Pharmacy, University of North Carolina Medical Center, Chapel Hill, NC, USA; Department of Clinical Pharmacy, University of Michigan College of Pharmacy, Ann Arbor, MI; Department of Pharmacy, Michigan Medicine, Ann Arbor, MI, USA

**Keywords:** cardiology, chronic kidney disease, clinical pharmacist, diabetes, finerenone, pharmacotherapy

## Abstract

**Purpose:**

This review provides an overview of the management of chronic kidney disease (CKD) associated with type 2 diabetes (T2D), how the novel treatment class of nonsteroidal mineralocorticoid receptor antagonists (MRAs) fits within the treatment landscape, and how pharmacists can contribute to the multidisciplinary care of patients with CKD associated with T2D.

**Summary:**

Optimizing pharmacotherapy for patients with CKD associated with T2D is critical to prevent or slow progression to end-stage kidney disease and reduce the incidence of cardiovascular events. However, many patients with CKD receive suboptimal treatment, in part because of the high complexity of care required, a lack of disease recognition among providers and patients, and a failure to utilize new kidney-protective therapies. Finerenone is the first nonsteroidal, selective MRA to be approved by the US Food and Drug Administration and the European Medicines Agency for the treatment of adult patients with CKD associated with T2D. Clinical trials have demonstrated that finerenone significantly reduces the risk of cardiorenal disease progression vs placebo and has a reduced risk of hyperkalemia compared to traditional steroidal MRAs. Initiation of finerenone should follow evaluation of baseline estimated glomerular filtration rate and serum potassium levels. Consideration of potential drug-drug interactions, follow-up monitoring of potassium levels, and coordination of changes in pharmacotherapy across the patient care team are also important.

**Conclusion:**

Finerenone is a valuable addition to the treatment landscape for CKD associated with T2D. Through their expertise in ­medication ­management, transitions of care, and patient education, clinical pharmacists are well positioned to ensure patients receive safe and effective ­treatment.

**KEY POINTS**

- Polypharmacy is common in chronic kidney disease (CKD) associated with type 2 diabetes (T2D), and the related challenges can lead to a failure to optimize pharmacotherapy and adopt new treatments for patients.
- Nonsteroidal mineralocorticoid receptor antagonist (MRA) therapy with finerenone significantly reduces cardiorenal risk, and expert guidelines recommend it for patients with an estimated glomerular filtration rate of at least 25 mL/min/1.73 m^2^ and normal serum potassium levels who have persistent albuminuria despite first-line interventions.
- Finerenone has a reduced risk of hyperkalemia compared to steroidal MRAs, which can be safely managed for most patients with appropriate monitoring and mitigation.

Chronic kidney disease (CKD) is a major contributor to global morbidity and mortality, with a reported prevalence of 9.1% in 2017.^1^ It is classified based on cause, rate of glomerular filtration (assessed by estimated glomerular filtration rate, or eGFR), and albuminuria category (assessed by urinary albumin-to-creatinine ratio, or UACR).^2,3^ In the US, the prevalence of CKD in the adult population was reported to range from 13% to 15% between 2003 and 2018 based on single examinations of eGFR or albuminuria.^4^ CKD is associated with large healthcare expenditures, with spending for beneficiaries with CKD accounting for approximately 30% of total Medicare fee-for-service spending in 2019.^4^

Hyperglycemia and hypertension are key contributors to the progression of kidney disease, and both are associated with type 2 diabetes (T2D).^5^ Consequently, the prevalence of CKD among patients with T2D in the US is between 35% and 40%, which is considerably higher than in the general population.^4,6^ Patients with CKD associated with T2D have high rates of progression to end-stage kidney disease (ESKD) and the accompanying burden of cardiovascular disease (CVD).^7^

CKD management requires a multifactorial approach encom­passing lifestyle and pharmacological interventions that necessitate the input and coordination of a multidisciplinary team (MDT).^8^ This is further complicated by high rates of comorbidities and polypharmacy.^8^ In addition, kidney disease can result in significant changes in the pharmacokinetic and pharmacodynamic properties of many medications.^9^ Integration of clinical pharmacists into the MDT can reduce the likelihood of medication therapy problems associated with these challenges.^9-11^

Early identification and initiation of interventions to prevent or slow disease progression are imperative, but many patients receive suboptimal treatment, in part because of a lack of disease recognition among providers and patients, the high complexity of care required, and a failure to utilize new kidney-protective therapies.^12^ The selective nonsteroidal mineralocorticoid receptor antagonist (nsMRA) finerenone is the latest kidney-protective agent to be approved by the US Food and Drug Administration (FDA) for the treatment of adult patients with CKD associated with T2D.^13^ It is indicated to reduce the risk of sustained eGFR decline, ESKD, cardiovascular death, nonfatal myocardial infarction, and hospitalization for heart failure.^14^ Finerenone has the most advanced development program of drugs in its class; however, several other nsMRAs are in development but have not been approved by FDA, including AZD9977, apararenone, KBP-5074, and esaxerenone.^13^

## Objectives

The aim of this review is to provide an overview of the management of CKD associated with T2D, with a focus on the role of finerenone within a rapidly changing treatment landscape. We will provide guidance on the safe and effective use of finerenone, including pharmacokinetics, dosing, risk assessment for and management of hyperkalemia, and potential drug-drug interactions (DDIs). Finally, we will consider how pharmacists can contribute to the multidisciplinary care of patients with CKD associated with T2D.

## Search strategy

PubMed was reviewed for articles published up until January 2023 that included the terms “finerenone,” “BAY94-8862,” and/or “hyperkalemia.” The references cited by pertinent articles were also searched for relevant publications.

## Diagnosis and clinical management of CKD associated with T2D

**Figure 1.**
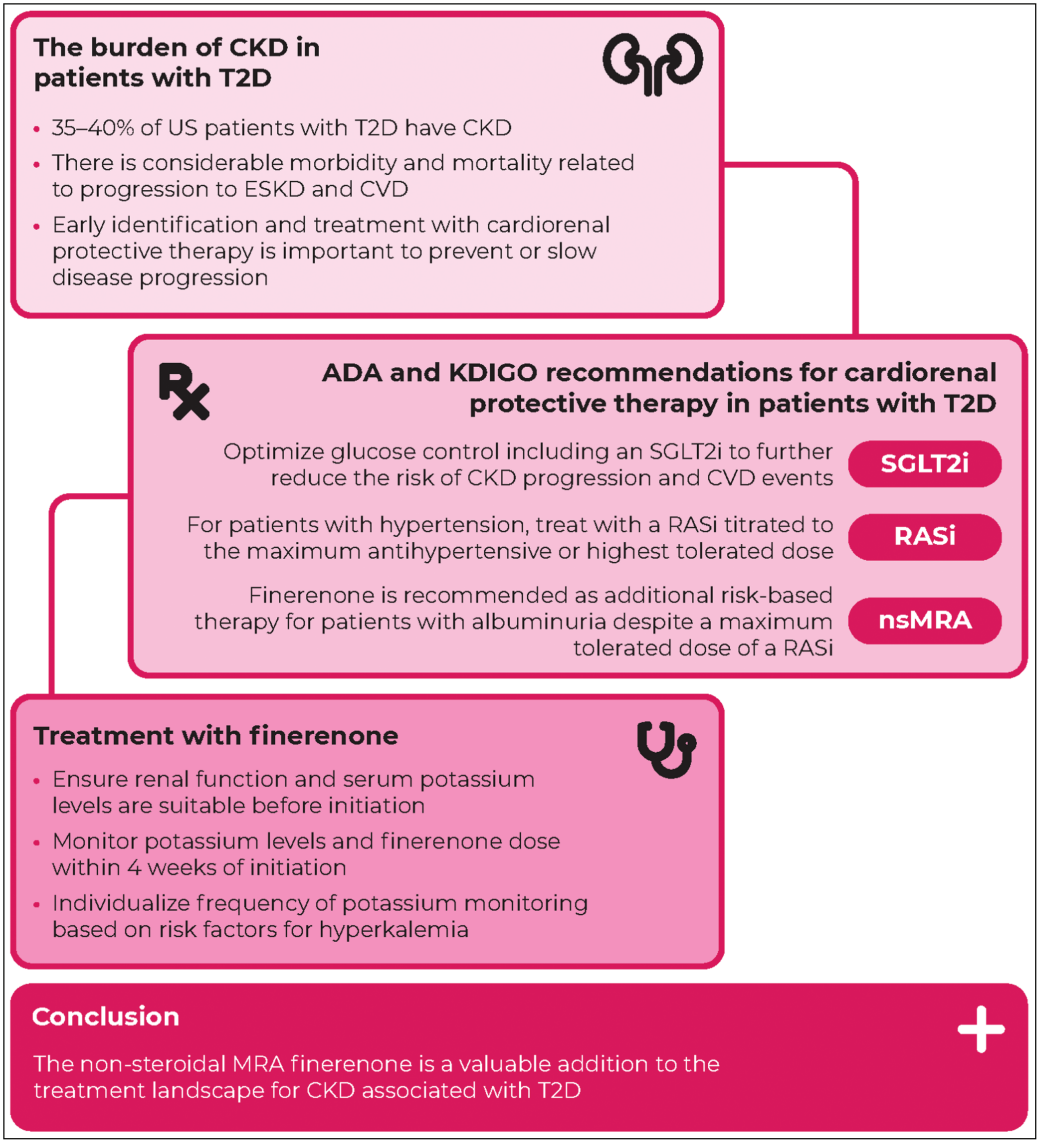
Graphical article summary. Finerenone is a nonsteroidal mineralocorticoid receptor antagonist (nsMRA) approved by the US Food and Drug Administration and European Medicines Agency to treat adult patients with chronic kidney disease (CKD) associated with type 2 diabetes (T2D). ACR indicates albumin-to-creatinine ratio; ADA, American Diabetes Association; CVD, cardiovascular disease; ESKD, end-stage kidney disease; KDIGO, Kidney Disease: Improving Global Outcomes; RASi, renin-angiotensin system inhibitor; SGLT2i, sodium-glucose cotransporter-2 inhibitor.

The American Diabetes Association (ADA) and the Kidney Disease: Improving Global Outcomes (KDIGO) organization, which issue separate guidelines for the management of CKD, recently released a consensus statement covering broad recommendations for management of CKD associated with T2D and specific guidance on the use of cardiorenal protective agents (summarized in Figure 1).^2,3,15^ The overall goals of disease management are to preserve organ function and attain intermediate targets for glycemia, blood pressure, and lipids through lifestyle interventions and pharmacological therapies if required.^15^ Recommended pharmacological therapies are summarized in Table 1.

**Table 1.** ADA and KDIGO Consensus Recommendations for Pharmacological Management of CKD Associated with T2D^15^

Drug or drug class	Effect	ADA and KDIGO consensus recommendations
Metformin	Glycemic control	First-line therapy for patients with an eGFR of ≥30 mL/min/1.73 m^2^; the dose should be reduced to 1,000 mg daily in patients with an eGFR of 30-44 mL/min/1.73 m^2^ and in some patients with an eGFR of 45-59 mL/min/1.73 m^2^ who are at high risk of lactic acidosis
SGLT2i	Glycemic control and cardiorenal protection	First-line therapy for patients with an eGFR of ≥20 mL/min/1.73 m²; if it has been initiated, the SGLT2i can be continued in patients with lower levels of eGFR
GLP-1RAs	Glycemic control and CVD protection	An additional risk-based therapy for patients who do not meet their individualized glycemic target with metformin and/or an SGLT2i or who are unable to use these drugs
Statins	Prevention of ASCVD	First-line therapy for all patients; moderate intensity for primary prevention of ASCVD or high intensity for patients with known ASCVD and some patients with multiple ASCVD risk factors
RAS inhibitors (ACE inhibitors and ARBs)	Antihypertensive	First-line therapy for patients who have hypertension and albuminuria, titrated to the maximum antihypertensive or highest tolerated dose
nsMRA (finerenone^a^)	Cardiorenal protection	An additional risk-based therapy for patients with an eGFR of ≥25 mL/min/1.73 m^2^, normal serum potassium concentration, and albuminuria (UACR of ≥30 mg/g) despite a maximum tolerated dose of a RAS inhibitor

Abbreviations: ACE, angiotensin-converting enzyme; ARB, angiotensin II receptor blocker; ASCVD, atherosclerotic cardiovascular disease; CKD, chronic kidney disease; eGFR, estimated glomerular filtration rate; GLP-1RA, glucagon-like peptide-1 receptor agonist; nsMRA, nonsteroidal mineralocorticoid receptor antagonist; RAS, renin-angiotensin system; SGLT2i, sodium-glucose cotransporter-2 inhibitor; T2D, type 2 diabetes; UACR, urinary albumin-to-creatinine ratio.

^a^Finerenone is the only nsMRA approved by the US Food and Drug Administration or European Medicines Agency for the treatment of CKD associated with T2D.

Sodium-glucose cotransporter-2 (SGLT2) inhibitors, metformin, statins, and renin-angiotensin system (RAS) inhibitors are recommended as first-line therapies. Metformin will usually form the basis of glycemic control, but it is contraindicated in patients with severely decreased eGFR (<30 mL/min/1.73 m^2^).^15^ SGLT2 inhibitors provide glycemic control as well as cardiorenal protection and may be used independently of metformin. The glucose-lowering effect of SGLT2 inhibitors is blunted at eGFRs of less than 45 mL/min/1.73 m², but these agents offer residual cardiorenal protection as eGFR declines to 25 mL/min/1.73 m².^2^

Statins are recommended for all patients with CKD associated with T2D for prevention or management of atherosclerotic CVD, and RAS inhibition with angiotensin-converting enzyme (ACE) inhibitors or angiotensin II receptor blockers (ARBs) is recommended for patients with hypertension and albuminuria.^15^ Despite being the standard of care for CKD associated with T2D for almost 20 years, ACE inhibitors and ARBs are widely underutilized, potentially because of concerns over complications such as acute kidney injury and hyperkalemia.^12^ A US registry study of data gathered from 2007 to 2017 reported that only 20.5% of all patients with CKD (N = 606,064) were prescribed an ACE inhibitor or ARB, and usage among patients with CKD and hypertension was only 25.9%.^16^ This highlights the challenges that exist in ensuring that patients with CKD receive optimal treatment.

Glucagon-like peptide-1 receptor agonists (GLP-1RAs) are recommended if additional risk factors are present. GLP-1RAs should be considered for patients who do not reach glycemic targets with metformin, which reflects the fact that most patients in clinical trials of GLP-1RAs have been on background metformin.^15,17^ GLP-1RAs also provide protection from CVD events. Positive outcomes for exploratory renal endpoints have been reported in several clinical trials, including reduced incidence of albuminuria and slowed eGFR decline, but the renal benefits of GLP-1RAs have not yet been definitively established within a clinical trial.^17^ Further insight will be provided by the ongoing phase 3 FLOW trial (primary completion date in August 2024), which will assess the impact of the GLP-1RA semaglutide on renal outcomes as a primary endpoint in patients with CKD associated with T2D.^2^

Steroidal MRAs, such as first-generation spironolactone and second-generation eplerenone, are indicated for the management of hypertension as well as heart failure with reduced ejection fraction, primary hyperaldosteronism (spironolactone only), and edema in cirrhosis (spironolactone only).^18,19^ They are not recommended in patients with severe kidney disease or ESKD, and eplerenone is contraindicated in patients with T2D and microalbuminuria because of an increased risk of hyperkalemia.^15,18,19^ The ADA and KDIGO consensus guidelines recommend steroidal MRAs for patients with an eGFR of at least 45 mL/min/1.73 m^2^ who have resistant hypertension despite previous treatment with a RAS inhibitor at the maximum tolerated dose as well as the calcium channel blocker dihydropyridine and/or a diuretic.^15^ The primary reason for the limited therapeutic role of steroidal MRAs is a risk of hyperkalemia, which is highest in patients with the most severely reduced filtration rates. Although there is some evidence that steroidal MRAs can improve renal outcomes for patients with kidney disease, data are very limited. To date, there have been no large-scale trials investigating renal outcomes in patients with diabetes treated with steroidal MRAs.

nsMRAs were developed to be highly selective for the MR, with the aim of providing cardiorenal protection with a tolerable adverse effect profile.^20^ Reflecting a reduced risk of hyperkalemia and definitive evidence for long-term cardiorenal protection in patients with CKD associated with T2D, finerenone is recommended for a less restricted subset of patients than the steroidal MRAs.^15^ Finerenone should be considered for patients with an eGFR of at least 25 mL/min/1.73 m^2^, normal serum potassium levels (≤5.0 mEq/L), and albuminuria (UACR of ≥30 mg/g).^15^ A further criterion for finerenone use is that patients are being treated with a maximum tolerated RAS inhibitor dose, which reflects the fact that the cardiorenal protection offered by finerenone in CKD associated with T2D has been demonstrated primarily in patients on a maximum tolerated dose of an ACE inhibitor or ARB.^15,21,22^

## Finerenone mechanism of action and preclinical data

Finerenone is a potent nsMRA with greater selectivity for the MR than the steroidal MRAs spironolactone and eplerenone.^20^ It has 500-fold-greater selectivity for the MR vs other steroid hormone receptors; by comparison, spironolactone and eplerenone had 3-fold- and 22-fold-greater selectivity, respectively, for the MR vs other steroid hormone receptors in the same assay.^20^

**Figure 2.**
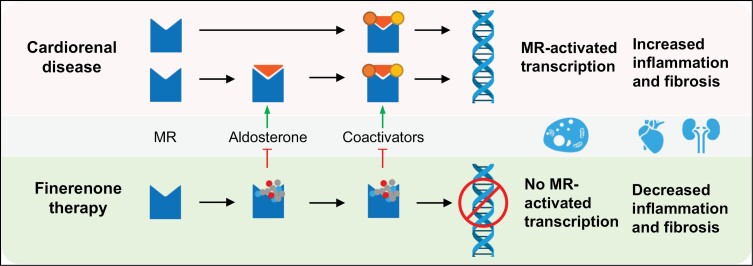
Mechanism of action and cardiorenal outcomes of finerenone.^14,27^ MR indicates mineralocorticoid receptor.

Preclinical studies have demonstrated that finerenone has a unique mechanism of action compared to spironolactone and eplerenone owing to its bulky structure.^20^ It completely inhibits MR coactivator binding in the presence of aldosterone, as well as in its absence, where it acts as an inverse agonist (Figure 2). In contrast, eplerenone partially inhibits aldosterone-mediated MR coactivator binding and induces coactivator binding in the absence of aldosterone.^23^ Finerenone has been shown to offer cardiorenal protection in animal models by reducing the fibrotic and inflammatory processes associated with overactivation of the MR.^23-25^

MRAs have the potential to increase potassium levels through inhibition of aldosterone-activated renal sodium reabsorption and potassium excretion.^26^ The risk of hyperkalemia associated with finerenone appears to be less than that with spironolactone and eplerenone, and this difference may relate to the tissue distribution of these agents. Animal studies have indicated that spironolactone and eplerenone may accumulate more in the kidneys than in the heart, whereas finerenone exhibits balanced accumulation in the heart and kidneys.^27^

## Clinical trials of finerenone in CVD and kidney disease

The efficacy and safety profiles of finerenone were evaluated in 3 phase 2 trials in patients with CVD and kidney disease within the ARTS program and 2 phase 3 trials in patients with CKD associated with T2D (Table 2).^21,22,28-30^

**Table 2.** Clinical Trial Outcomes of Finerenone in Patients With Cardiorenal Disease

Trial	Study design	Key inclusion/exclusion criteria	Treatments	Primary endpoint(s)	Secondary and/or safety endpoints^a^
ARTS^28^	Phase 2, two-part study of the safety and tolerability of finerenone in patients with HFrEF associated with mild or moderate CKD. Duration: Patients received the study drug for 4 weeks, with final assessment 2 weeks after last intake of the drug. • Part A: Safety of finerenone in patients with mild CKD (n = 65) • Part B: Safety and efficacy of finerenone vs placebo or spironolactone in patients with moderate CKD (n = 389)	Inclusion: Receiving evidence-based therapy for HFrEF, serum potassium of ≤4.8 mEq/L, eGFR of 60 to <90 mL/min/1.73 m² in part A or 30-60 mL/min/1.73 m² in part B. Exclusion: Use of a renin inhibitor or an aldosterone antagonist 30 days before randomization, worsening heart failure requiring hospitalization, and IV diuretics within 30 days of screening visit (if not on an aldosterone antagonist) or 6 months before screening visit (if receiving an aldosterone antagonist immediately before the study).	• Part A: Finerenone (2.5, 5, or 10 mg once daily) vs placebo • Part B: Finerenone (2.5, 5, or 10 mg once daily or 5 mg twice daily) vs spironolactone (25 or 50 mg once daily) vs placebo	• Part A: The safety and tolerability of finerenone were confirmed by an independent data monitoring committee. • Part B: Finerenone 10 mg once daily and 5 mg twice daily increased serum potassium concentration from baseline at the study endpoint vs placebo (*P* = 0.0243 and *P* = 0.0003, respectively).	• Finerenone at doses of 5 and 10 mg once daily decreased the levels of BNP and NT-pro-BNP and albuminuria to at least the same extent as spironolactone 25 or 50 mg once daily. • Mean increases in serum potassium concentrations were lower with finerenone than with spironolactone (0.04-0.30 vs 0.45 mEq/L; *P* < 0.0001-0.0107).
ARTS-HF^29^	Phase 2b, randomized, double-blind, parallel-group dose-finding trial in adult patients (N = 1,066) with worsening HFrEF requiring hospitalization and treatment with IV diuretics. Duration: Patients received the study drug for 90 days, with final assessment 30 days after last intake of the drug.	Inclusion: T2D and/or CKD (eGFRs of >30 mL/min/1.73 m² in patients with CKD and 30-60 mL/min/1.73 m² in patients without CKD), receiving evidence-based therapy for heart failure for ≥3 months, and LVEF of ≤40% in past 12 months. Exclusion: Patients receiving spironolactone, eplerenone, renin inhibitors, or potassium-sparing diuretics who were unable to discontinue them for the study period.	Finerenone (2.5-5, 5-10, 7.5-15, 10-20, or 15-20 mg once daily) vs eplerenone (25 mg every other day increased to 25 mg once daily on day 30 and to 50 mg once daily on day 60)	The proportion of patients with a decrease of >30% in plasma NT-pro-BNP level from baseline was similar between the finerenone groups and eplerenone. All doses of finerenone had safety outcomes similar to those with eplerenone.	A composite clinical endpoint of death from any cause, cardiovascular hospitalization, and emergency presentation for worsening heart failure occurred less frequently in all the finerenone groups except the group receiving 2.5-5 mg once daily vs eplerenone. The difference was nominally significant in the group receiving 10-20 mg (HR, 0.56; 95% CI, 0.35-0.90; *P* = 0.02), although the study was not powered to detect statistically significant differences.
ARTS-DN^30^	Phase 2b, randomized, double-blind, parallel-group dose-finding trial in adult patients (N = 821) with T2D and persistent albuminuria (UACR of ≥30 mg/g). Duration: Patients received the study drug for 90 days, with final assessment 30 days after last intake of the drug.	Inclusion: eGFR of >30 mL/min/1.73 m², at least the minimum label-recommended dose of an ACEi or ARB, and serum potassium of ≤4.8 mEq/L. If eGFR was 30-45 mL/min/1.73 m², the patient must have been receiving a non–potassium-sparing diuretic for ≥4 weeks without dose adjustment. Exclusion: Treatment with spironolactone, eplerenone, or a renin inhibitor.	Finerenone (1.25, 2.5, 5, 7.5, 10, 15, or 20 mg once daily) vs placebo for 90 days. (ACEi or ARB dose at baseline: 68.4% of patients on a dose above the minimum and up to the maximum label-recommended dose and 4.3% on a dose higher than the maximum label-recommended dose).^b^	Finerenone demonstrated a dose-dependent reduction in UACR at day 90 vs baseline (one-sided *F* test for linear contrast, *P* < 0.001). The placebo-corrected mean ratio of the UACR at day 90 relative to baseline was reduced in the finerenone 7.5, 10, 15, and 20 mg once daily groups (7.5 mg: 0.79; 90% CI, 0.68-0.91; *P* = 0.004; 10 mg: 0.76; 90% CI, 0.65-0.88; *P* = 0.001; 15 mg: 0.67; 90% CI, 0.58-0.77; *P* < 0.001; 20 mg: 0.62; 90% CI, 0.54-0.72; *P* < 0.001). A placebo-corrected reduction in UACR in the groups receiving 7.5, 10, 15, and 20 mg once daily (7.5 mg: 0.79; 90% CI, 0.68-0.91; *P* = 0.004; 10 mg: 0.76; 90% CI, 0.65-0.88; *P* = 0.001; 15 mg: 0.67; 90% CI, 0.58-0.77; *P* < 0.001; 20 mg: 0.62; 90% CI, 0.54-0.72; *P* < 0.001).	Hyperkalemia leading to discontinuation occurred in the finerenone groups receiving 7.5, 15, and 20 mg once daily at a rate of 2.1%, 3.2%, and 1.7%, respectively. No difference was observed between the groups in eGFR reduction of ≥30%.
FIDELIO-DKD^22^	Phase 3, randomized, double-blind trial in adult patients with CKD associated with T2D receiving an ACEi or ARB (N = 5,734). Median follow-up: 2.6 years.	Inclusion: UACR of 30 to <300 mg/g, eGFR of 25 to <60 mL/min/1.73 m², and history of diabetic retinopathy; or UACR of 300-5,000 mg/g, eGFR of 25 to <75 mL/min/1.73 m², serum potassium of ≤4.8 mEq/L, and on a maximum tolerated dose of an ACEi or ARB. Exclusion: Nondiabetic kidney disease, uncontrolled hypertension, HbA_1c_ of >12%, HFrEF (NYHA class II-IV), and already taking eplerenone, spironolactone, a renin inhibitor, or a potassium-sparing diuretic.	Finerenone (starting at 10 to 20 mg once daily depending on renal function) vs placebo. (ACEi or ARB dose at baseline: 68.6% of patients on a dose above the minimum and up to the maximum label-recommended dose and 1.0% on a dose higher than the maximum label-recommended dose).^b^	Incidence of the primary composite outcome comprising kidney failure, a sustained decrease of at least 40% in eGFR from baseline over a period of ≥4 weeks, and death from renal causes was reduced with finerenone vs placebo (17.8% vs 21.1%; HR, 0.82; 95% CI, 0.73-0.93; *P* = 0.001).	Incidence of the key secondary composite outcome comprising death from cardiovascular causes, nonfatal MI, nonfatal stroke, and hospitalization for heart failure was reduced with finerenone vs placebo (13.0% vs 14.8%; HR, 0.86; 95% CI, 0.75-0.99; *P* = 0.03).
FIGARO-DKD^21^	Phase 3, randomized, double-blind trial in adult patients (N = 5,734) with CKD associated with T2D receiving an ACEi or ARB. Median follow-up: 3.4 years.	Inclusion: UACR of 30 to <300 mg/g and eGFR of 25-90 mL/min/1.73 m²; or UACR of 300-5,000 mg/g, eGFR of ≥60 mL/min/1.73 m², serum potassium of ≤4.8, and on a maximum tolerated dose of an ACEi or ARB. Exclusion: UACR of 300-5,000 mg/g and eGFR of 25 to <60 mL/min/1.73 m²; or nondiabetic kidney disease; or uncontrolled hypertension; or HbA_1c_ of >12%; or HFrEF (NYHA class II-IV); or already taking eplerenone, spironolactone, a renin inhibitor, or a potassium-sparing diuretic.	Finerenone (starting at 10 mg once daily and increasing to 20 mg once daily) vs placebo. (ACEi or ARB dose at baseline: 68.6% of patients on a dose above the minimum and up to the maximum label-recommended dose and 0.7% on a dose higher than the maximum label-recommended dose).^b^	Incidence of the primary composite outcome comprising death from cardiovascular causes, nonfatal MI, nonfatal stroke, and hospitalization for heart failure was reduced with finerenone vs placebo (12.4% vs 14.2%; HR, 0.87; 95% CI, 0.76-0.98; *P* = 0.03).	Incidence of the key secondary composite outcome comprising kidney failure, a sustained decrease of at least 40% in eGFR from baseline over a period of ≥4 weeks, and death from renal causes was not significantly different for finerenone vs placebo (9.5% vs 10.8%; HR, 0.87; 95% CI, 0.76-1.01).

Abbreviations: ACEi, angiotensin-converting enzyme inhibitor; ARB, angiotensin II receptor blocker; BNP, B-type natriuretic peptide; CI, confidence interval; CKD, chronic kidney disease; eGFR, estimated glomerular filtration rate; HbA_1c_, glycated hemoglobin; HFrEF, heart failure with reduced ejection fraction; HR, hazard ratio; IV, intravenous; LVEF, left ventricular ejection fraction; MI, myocardial infarction; NT-pro-BNP, N-terminal pro-B-type natriuretic peptide; NYHA, New York Heart Association; T2D, type 2 diabetes; UACR, urinary albumin-to-creatinine ratio.

^a^Secondary endpoints in the phase 2 trials were exploratory; no adjustments were made for multiple comparisons.

^b^Data provided within supplementary appendices to the cited publications.

The phase 2a ARTS study established safe dosing of finerenone. Assessment of exploratory endpoints suggested that finerenone offered equivalent or greater reductions in albuminuria and N-terminal pro-B-type natriuretic peptide levels than spironolactone, as well as a lower incidence of hyperkalemia.^28^ The cardiorenal effects of finerenone are not mediated through antihypertensive effects, as evidenced by minimal effects on blood pressure compared to spironolactone and no evidence of a correlation between the antialbuminuric effects of finerenone and blood pressure changes.^28^

The phase 2b ARTS-HF trial in patients with worsening heart failure who also had T2D and/or CKD demonstrated similar effects of finerenone and eplerenone on N-terminal pro-B-type natriuretic peptide levels and similar safety profiles. The mean change in blood pressure was similar with finerenone and eplerenone.^29^ The phase 2b ARTS-DN trial demonstrated a dose-dependent reduction in UACR with finerenone in patients with CKD associated with T2D who were being treated with an ACE inhibitor or ARB.^30^

Finerenone was next compared to the standard of care in patients with CKD associated with T2D stabilized on the maximum tolerated dose of an ACE inhibitor or ARB in the phase 3, randomized, double-blind, placebo-controlled FIDELIO-DKD and FIGARO-DKD trials.^21,22^ The trials had reciprocal primary and secondary endpoints, and the patient populations in each study reflected the focus on renal (FIDELIO-DKD) or CVD (FIGARO-DKD) outcomes.^26^ The FIDELIO-DKD trial had a primary composite endpoint of renal outcomes and a secondary composite endpoint of CVD outcomes, both of which were met.^22^ In the FIGARO-DKD trial, CVD outcomes made up the primary composite endpoint and the secondary composite endpoint comprised renal outcomes.^21^

In the FIGARO-DKD trial, the primary composite endpoint was met but not the secondary composite endpoint.^21^ In a prespecified patient-level pooled analysis (FIDELITY) of the outcomes from the FIDELIO-DKD and FIGARO-DKD trials, the incidence of both kidney failure and CVD events was significantly reduced with finerenone compared to placebo, although the CVD benefit was predominantly derived from a reduced incidence of hospitalizations for heart failure.^31^

Across the FIDELIO-DKD and FIGARO-DKD trials, adverse events (AEs) related to treatment occurred in 18.5% of patients treated with finerenone vs 13.3% of those treated with placebo, and AEs leading to treatment discontinuation occurred in 6.4% vs 5.4% of patients, respectively.^31^ Investigator-reported hyperkalemia was more frequent with finerenone (14%) than with placebo (6.9%); incidence of permanent treatment discontinuation due to hyperkalemia was low across study arms but occurred more frequently with finerenone (1.7%) than with placebo (0.6%). No hyperkalemia-related AEs were fatal.^31^

## Place in therapy

Cardiorenal protection with finerenone is supported by both the ADA and KDIGO guidelines in patients with CKD and T2D.^2,3,15^ Appropriate patient selection is crucial, with special attention to ensuring that first-line therapies of metformin, SGLT2 inhibitors, maximum tolerated doses of RAS inhibitors (for hypertensive patients), and moderate- or high-intensity statins have been trialed. Finerenone should be considered for patients with an eGFR of at least 25 mL/min/1.73 m^2^ and normal serum potassium levels (≤5.0 mEq/L) who have persistent albuminuria (UACR of ≥30 mg/g) despite first-line interventions.^14,15^ Finerenone may also be considered as an add-on therapy to a RAS inhibitor for patients who do not tolerate or are ineligible for an SGLT2 inhibitor.^3^

## Future outlook

It will be important to define where finerenone best fits within the treatment landscape for CKD associated with T2D, particularly in relation to SGLT2 inhibitors and GLP-1RAs. Further research is needed to determine the relative effects of these agents and classes in different patient populations and whether they should be used in combination. This is an interesting concept because these agents have different mechanisms of action and may, therefore, have the potential to provide additive or synergistic treatment effects.

SGLT2 inhibitors provide cardiorenal protection and help to improve glycemic control, but their glucose-lowering effects are blunted in patients with an eGFR of below 45 mL/min/1.73 m².^2^ For these patients, additional studies are needed to determine whether cardiorenal protection is best provided by an SGLT2 inhibitor, finerenone, or use of both therapies. An exploratory analysis of patients receiving an SGLT2 inhibitor at baseline who were treated with finerenone (n = 124) in the FIDELIO-DKD trial reported numerically lower rates of hyperkalemia-related AEs and greater reductions in UACR compared to those not receiving an SGLT2 inhibitor at baseline (n = 2,703).^32^

Further clarity is anticipated from the prospective phase 2 CONFIDENCE study.^33^ In this ongoing study, approximately 807 patients with CKD associated with T2D (eGFR of 30-90 mL/min/1.73 m^2^) were randomized to receive monotherapy with finerenone, monotherapy with the SGLT2 inhibitor empagliflozin, or combination therapy with finerenone and empagliflozin. With an estimated primary completion date in November 2023, the CONFIDENCE study aims to determine whether dual therapy with finerenone and an SGLT2 inhibitor is superior to treatment with either agent alone. Additionally, analysis of the outcomes for patients in the 2 monotherapy arms may provide some answers as to the relative cardiorenal protection provided by finerenone vs empagliflozin at lower levels of kidney function.^34^

With regard to GLP-1RAs, a post hoc exploratory analysis of the FIDELIO-DKD trial found no evidence for an increased benefit of baseline GLP-1RA use in patients who were treated with finerenone (n = 189) compared to patients treated with finerenone without baseline GLP-1RA use (n = 2,638).^35^ At the time of writing, no prospective clinical trials were being planned to evaluate the combination of finerenone and GLP-1RAs in patients with CKD associated with T2D.

## Pharmacotherapy considerations for finerenone

### Pharmacokinetics.

Finerenone is administered orally and is rapidly absorbed, with the maximum serum concentration (*C*_max_) reached between 0.5 and 1.25 hours after dosing.^14^ Absolute bioavailability is approximately 44%, predominantly because of first-pass metabolism by cytochrome P450 family 3 subfamily A member 4 (CYP3A4) in the gut wall and liver.^14,36^ Food does not have a clinically significant impact on finerenone bioavailability, although high-fat and high-calorie foods affect the rate of absorption.^14,37^

Finerenone is metabolized to inactive metabolites by CYP3A4 (~90%) and CYP2C8, and approximately 80% of the administered dose is eliminated in urine, with the remainder eliminated in feces.^14^ The terminal half-life is between 2 and 3 hours, and systemic blood clearance is 25 L/h.^14^

Mild renal impairment has no clear effect on finerenone bioavailability or *C*_max_. Moderate hepatic impairment, however, results in a 38% increase in finerenone area under the plasma concentration–time curve (AUC) and a 55% increase in the AUC of unbound finerenone compared to healthy individuals, with no change in *C*_max_.^38^

### Dosing considerations.

**Figure 3.**
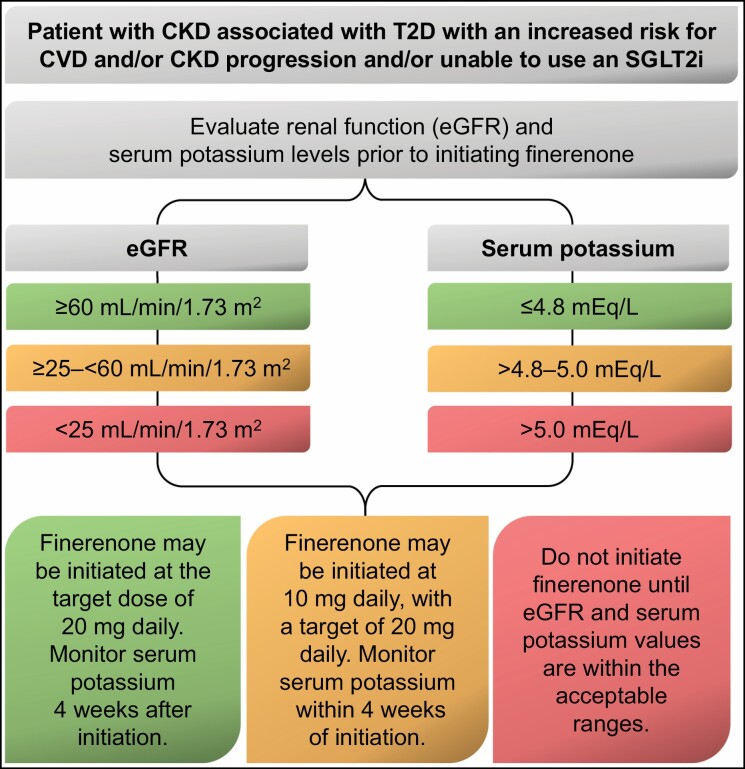
Dose selection for initiation of finerenone.^14^ CKD indicates chronic kidney disease; CVD, cardiovascular disease; eGFR, estimated glomerular filtration rate; SGLT2i, sodium-glucose cotransporter-2 inhibitor; T2D, type 2 diabetes.

Finere­none is available in 10- and 20-mg film-coated tablets, with a target dose of 20 mg once daily.^14^ The recommended starting dose is dependent on eGFR and is 20 mg once daily for patients with normal or mildly reduced eGFR (≥60 mL/min/1.73 m²), which is reduced to 10 mg once daily for those with more severe renal impairment (25 to 59 mL/min/1.73 m²); initiation is avoided in patients with an eGFR of below 25 mL/min/1.73 m² (Figure 3).^14^

The tablets, which are not scored, may be crushed and administered orally with water or soft foods for patients unable to swallow them whole.^14^ A missed dose should be taken as soon as possible but only on the day it was missed.^14^

### Hyperkalemia: risk assessment and management.

Serum potassium levels should be evaluated before initiation of finerenone (Figure 3). Treatment should not be initiated if the serum potassium concentration is above 5.0 mEq/L, but initiation may be considered if the concentration is greater than 4.8 but not above 5.0 mEq/L based on clinical judgment, with close monitoring of potassium levels within the first 4 weeks.^14^ For patients with a baseline serum potassium level of 4.8 mEq/L or less, repeat potassium measurement should be performed within 4 weeks of finerenone initiation and the finerenone dose should be adjusted if the serum potassium concentration is more than 4.8 mEq/L but less than or equal to 5.5 mEq/L. Finerenone should be withheld if the serum potassium concentration is more than 5.5 mEq/L and restarted at 10 mg once daily only when levels are 5.0 mEq/L or less.^14^

Closer monitoring is recommended for patients with high baseline potassium levels and a low eGFR, as well as other risk factors such as the presence of CVD and the potential for DDIs.^39^ Patients with CKD associated with T2D are likely to be on other medications associated with a risk of hyperkalemia at initiation of finerenone, such as an ACE inhibitor or ARB.^2,3^ Use of medications associated with a risk of hypokalemia, such as loop and thiazide diuretics, must also be considered because dose adjustments or cessation of these medications may result in fluctuations in serum potassium levels.^40^ Additional risk factors such as male sex, race, and diet are unlikely to affect the management of hyperkalemia in isolation but may have additive effects.^39^

In clinical practice, it is important to be aware of the potential for variability arising from methodological factors, particularly for patients in the window where clinical judgment may be exercised (with a serum potassium concentration of >4.8 to 5.0 mEq/L).^14,39^ Poor phlebotomy technique may cause pseudohyperkalemia due to hemolysis; excessive patient fist clenching during blood draws can have the same outcome through hypoxia-induced elevation of serum potassium levels. Potassium levels also fluctuate with circadian rhythm by about 0.60 mEq/L throughout the day, so the timing of blood draws may need to be considered with borderline results.^41^

Management of hyperkalemia differs between chronic and acute cases.^39^ The objective in management of acute hyperkalemia is to prevent or reduce the risk of cardiac arrhythmia, whereas treatment of chronic hyperkalemia, which is likely to be detected in routine monitoring and is often asymptomatic, is aimed at normalizing or reducing potassium levels to prevent acute hyperkalemia.^39^

Patients with chronic hyperkalemia should be advised to moderate dietary potassium intake and to avoid other sources of potassium, such as supplements and salt substitutes^3,39^ Working with a renal dietitian as part of the MDT may be helpful. Medications should be reviewed with dose optimization considered for RAS inhibitors.^3,39^ The risks associated with hyperkalemia should be weighed against the risk of downtitration or withdrawal of RAS inhibitors.^42^ In the case of ACE inhibitors and ARBs, it is important to consider that a majority of patients in the pivotal trials (70% in FIDELIO-DKD and 69% in FIGARO-DKD) were receiving an ACE inhibitor or ARB dose above the minimum label-recommended dose at baseline.^21,22^ Therefore, withdrawing or interrupting treatment with finerenone may be favored over adjustment of the ACE inhibitor or ARB dose to the minimum label-recommended dose in a situation where hyperkalemia requires management.

Use of diuretics and sodium bicarbonate may also be considered.^3,39^ Potassium binders may be used as well, particularly during optimization of the RAS inhibitor dose.^3,39^ Newer potassium binders such as patiromer and sodium zirconium cyclosilicate are associated with a reduced risk of gastrointestinal symptoms compared to sodium polystyrene sulfonate and calcium polystyrene sulfonate.^43^

### DDIs and drug-food interactions with finerenone.

The risk of DDIs is primarily influenced by agents that alter metabolism of finerenone, inhibit the RAS system, or increase serum potassium levels (Table 3). The main implication of DDIs and drug-food interactions associated with finerenone is an increased risk of hyperkalemia.^14^

**Table 3.** Potential Drug-Drug Interactions With Concomitant Finerenone^14,44,45^

Interacting drug or drug class	Effect	Recommendations
CYP3A4 inhibitors (eg, verapamil or diltiazem)	Increased exposure of finerenone	• Avoid strong CYP3A4 inhibitors • For moderate CYP3A4 inhibitors, monitor serum potassium during drug initiation and consider dose adjustments for finerenone or other agent
CYP3A4 inducers (eg, carbamazepine, modafinil, phenytoin, rifampin, or St John’s wort)	Reduced exposure of finerenone	Avoid moderate to strong CYP3A4 inducers
Spironolactone or eplerenone	Hyperkalemia caused by MR blockade	Spironolactone and eplerenone are not recommended for CKD associated with T2D and should be avoided in patients receiving finerenone
Aliskiren, ACE inhibitors, ARBs, or ARNIs	Hyperkalemia caused by impaired renin-angiotensin signaling	• Monitor serum potassium and consider dose adjustments for finerenone or other agent in the event of hyperkalemia • Aliskiren use should be avoided as patients taking renin inhibitors were excluded from the FIDELIO-DKD and FIGARO-DKD clinical trials
NSAIDs	Hyperkalemia caused by impaired renin production	• Monitor serum potassium and consider dose adjustments for finerenone or other agent in the event of hyperkalemia • NSAID use in patients with CKD is not recommended because of associated adverse renal events
Heparin or ketoconazole	Hyperkalemia caused by impaired aldosterone synthesis	• Monitor serum potassium and consider dose adjustments for finerenone or other agent in the event of hyperkalemia • Ketoconazole is a strong CYP3A4 inhibitor and should be avoided
Amiloride, triamterene, trimethoprim, or lithium	Hyperkalemia caused by epithelial sodium channel blockade	Monitor serum potassium and consider dose adjustments for finerenone or other agent in the event of hyperkalemia
Calcineurin inhibitors	Hyperkalemia caused by activation of the renal sodium chloride cotransporter	Monitor serum potassium and consider dose adjustments for finerenone or other agent in the event of hyperkalemia
α-agonists, β-blockers, digoxin, minoxidil, or somatostatin analogs	Hyperkalemia caused by changes in transcellular transporters	• Monitor serum potassium and consider dose adjustments for finerenone or other agent in the event of hyperkalemia • No clinically significant pharmacokinetic differences for finerenone with concomitant digoxin
Potassium-containing agents (eg, penicillin V potassium)	Hyperkalemia caused by high potassium content	Monitor serum potassium closely during concomitant therapy

Abbreviations: ACE, angiotensin-converting enzyme; ARBs, angiotensin II receptor blockers; ARNIs, angiotensin receptor–neprilysin inhibitors; CKD, chronic kidney disease; CYP3A4, cytochrome P450 family 3 subfamily A member 4; MR, mineralocorticoid receptor; NSAIDs, nonsteroidal anti-inflammatory drugs; T2D, type 2 diabetes.

Finerenone is primarily metabolized by CYP3A4, and concomitant use of strong CYP3A inhibitors is contraindicated because of the resultant increase in finerenone exposure.^14^ Concomitant use of the strong CYP3A inhibitor itraconazole increased the AUC of finerenone by more than 400%.^14^ Conversely, strong or moderate CYP3A inducers decrease finerenone exposure and may reduce the efficacy of finerenone.^14^ No clinically significant differences in finerenone pharmacokinetics have been measured with CYP2C8 inhibitors.^14^

## Special populations

No differences in safety or efficacy have been observed in geriatric populations compared to other adult populations.^14^ Safety and efficacy of finerenone have not been established in pediatric patients.^14^

Use of finerenone should be avoided in patients with severe hepatic impairment (Child-Pugh class C) but may be used without dose adjustment in patients with mild or moderate impairment (Child-Pugh class A or B). Additional serum potassium monitoring should be considered in patients with moderate impairment (Child-Pugh class B).^14^

There are no data on fetal or maternal outcomes with finerenone use during pregnancy. Animal studies have shown developmental toxicity at exposure levels 4 times those expected in humans.^14^ Similarly, there are no safety data for human breastfeeding, although animal studies have suggested that finerenone is likely to be present in milk. Consequently, it is recommended to avoid breastfeeding during treatment and for 1 day after treatment.^14^

## The role of the pharmacist

Clinical pharmacists play an important role in the management of patients with CKD, and evidence favors their involvement in the MDTs that coordinate patient care.^9-11^ Pharmacists offer valuable expertise in comprehensive medication management, transitions of care, and patient education.^9-11^ Communication among established members of the MDT within the areas of primary care, endocrinology, nephrology, and cardiology is important for optimal patient care.

Pharmacists are key to medication selection, optimization, and monitoring, in addition to patient education and counseling, and are essential in helping patients to overcome access and cost barriers. There may also be a daunting list of recommendations associated with prescribed medications, and pharmacists can help to ensure patients understand any mitigation measures and are reassured of the efficacy and safety of their medications. It is especially important that patients be counseled to report any concerns when they arise.

Suboptimal medication adherence is one of the largest problems in healthcare and is highly relevant in CKD associated with T2D, where many patients have a high medication burden and comorbidities.^11^ Nonadherence results from intentional factors, such as patient beliefs and priorities, and nonintentional factors, such as lack of education, poor organization, and forgetfulness. Pharmacists have expertise in addressing these issues through effective communication with patients and the introduction of various resources, including pill boxes and mobile phone applications.^9-11^

Finally, pharmacists may be involved with cost-benefit assessment for medications. Early modeling of the use of finerenone treatment in addition to standard of care in CKD associated with T2D predicted a significant cost benefit derived primarily through a reduced risk of ESKD and renal death in advanced CKD.^46^ Recently, the FINE-CKD model has been developed, which can be used to produce reliable assessments of the benefits and costs of the use of finerenone in patients with CKD associated with T2D.^47^

It is important to note that finerenone and the steroidal MRAs have clearly defined and exclusive roles within the ADA and KDIGO guidelines.^2,3,15^ Steroidal MRAs are recommended as antihypertensives for a more restricted subset of patients than finerenone, which is recommended for cardiorenal protection.^15^ Additionally, steroidal MRAs are not recommended in patients with severe kidney disease or ESKD, and eplerenone is contraindicated in patients with T2D and microalbuminuria.^18,19^ Therefore, steroidal MRAs should not be considered substitutes for finerenone in cases where there are cost or access issues.

## Conclusion

Finerenone provides cardiorenal protection for patients with CKD associated with T2D through effects on inflammatory and fibrotic pathways distinct from those of other currently approved treatments. An increased risk of hyperkalemia is a limitation of traditional steroidal MRAs, but preclinical and clinical data suggest that this risk is reduced with the nsMRA finerenone and is manageable for most patients. Clinical pharmacists have an opportunity to take a central role in ensuring prescribing practices deliver the benefits of the most effective available treatments to patients living with CKD associated with T2D.

## References

[1] GBD Chronic Kidney Disease Collaboration. Global, regional, and national burden of chronic kidney disease, 1990-2017: a systematic analysis for the Global Burden of Disease Study 2017. Lancet. 2020;395(10225):709-733. doi:10.1016/S0140-6736(20)30045-3 32061315 PMC7049905

[2] ElSayed NA , Aleppo G, Aroda VR, et al. 11. Chronic kidney disease and risk management: standards of care in diabetes—2023. Diabetes Care. 2023;46(suppl 1):S191-S202. 36507634 10.2337/dc23-S011 PMC9810467

[3] Kidney Disease: Improving Global Outcomes (KDIGO) Diabetes Work Group. KDIGO 2022 Clinical practice guideline for diabetes management in chronic kidney disease. Kidney Int. 2022;102(5S):S1-S127. doi:10.1016/j.kint.2022.06.008 36272764

[4] US Renal Data System. 2021 USRDS Annual Data Report: Epidemiology of Kidney Disease in the United States. US Renal Data System; 2021. https://adr.usrds.org/2021

[5] DeFronzo RA , Reeves WB, Awad AS. Pathophysiology of diabetic kidney disease: impact of SGLT2 inhibitors. Nat Rev Nephrol. 2021;17(5):319-334. doi:10.1038/s41581-021-00393-8 33547417

[6] Wu B , Bell K, Stanford A, et al. Understanding CKD among patients with T2DM: prevalence, temporal trends, and treatment patterns—NHANES 2007–2012. BMJ Open Diabetes Res Care. 2016;4(1):e000154. doi:10.1136/bmjdrc-2015-000154 PMC4838667 27110365

[7] Rangaswami J , Bhalla V, de Boer IH, et al. Cardiorenal protection with the newer antidiabetic agents in patients with diabetes and chronic kidney disease: a scientific statement from the American Heart Association. Circulation. 2020;142(17):e265-e286. doi:10.1161/CIR.0000000000000920 32981345

[8] Shubrook JH , Neumiller JJ, Wright E. Management of chronic kidney disease in type 2 diabetes: screening, diagnosis and treatment goals, and recommendations. Postgrad Med. 2022;134(4):376-387. doi:10.1080/00325481.2021.2009726 34817311

[9] Meaney CJ , Manley HJ, Pai AB, Battistella M, Hudson JQ, St. Peter WL. Nephrology practice and research network opinion paper: pharmacists’ perspectives on the Advancing American Kidney Health initiative. J Am Coll Clin Pharm. 2020;3(7):1355-1368. doi:10.1002/jac5.1309

[10] Al Raiisi F , Stewart D, Fernandez-Llimos F, Salgado TM, Mohamed MF, Cunningham S. Clinical pharmacy practice in the care of chronic kidney disease patients: a systematic review. Int J Clin Pharm. 2019;41(3):630-666. doi:10.1007/s11096-019-00816-4 30963447 PMC6554252

[11] St Peter WL , Wazny LD, Patel UD. New models of chronic kidney disease care including pharmacists: improving medication reconciliation and medication management. Curr Opin Nephrol Hypertens. 2013;22(6):656-662. doi:10.1097/MNH.0b013e328365b364 24076556 PMC4012859

[12] Neumiller JJ , Alicic RZ, Tuttle KR. Overcoming barriers to implementing new therapies for diabetic kidney disease: lessons learned. Adv Chronic Kidney Dis. 2021;28(4):318-327. doi:10.1053/j.ackd.2021.02.001 34922688

[13] Kintscher U , Bakris GL, Kolkhof P. Novel non-steroidal mineralocorticoid receptor antagonists in cardiorenal disease. Br J Pharmacol. 2022;179(13):3220-3234. doi:10.1111/bph.15747 34811750

[14] Kerendia (finerenone). Package insert. Bayer HealthCare Pharmaceuticals; 2021.

[15] de Boer IH , Khunti K, Sadusky T, et al. Diabetes management in chronic kidney disease: a consensus report by the American Diabetes Association (ADA) and Kidney Disease: Improving Global Outcomes (KDIGO). Kidney Int. 2022;102(5):974-989. doi:10.1016/j.kint.2022.08.012 36202661

[16] Tuttle KR , Alicic RZ, Duru OK, et al. Clinical characteristics of and risk factors for chronic kidney disease among adults and children: an analysis of the CURE-CKD Registry. JAMA Netw Open. 2019;2(12):e1918169. doi:10.1001/jamanetworkopen.2019.18169 31860111 PMC6991307

[17] ElSayed NA , Aleppo G, Aroda VR, et al. 10. Cardiovascular disease and risk management: Standards of Care in Diabetes—2023. Diabetes Care. 2023;46(suppl 1):S158-S190. 10.2337/dc23-S010 PMC9810475 36507632

[18] Inspra (eplerenone). Package insert. Pfizer; 2020.

[19] Aldactone (spironolactone). Package insert. Pfizer; 2022.

[20] Pitt B , Filippatos G, Gheorghiade M, et al. Rationale and design of ARTS: a randomized, double-blind study of BAY 94-8862 in patients with chronic heart failure and mild or moderate chronic kidney disease. Eur J Heart Fail. 2012;14(6):668-675. doi:10.1093/eurjhf/hfs061 22562554

[21] Pitt B , Filippatos G, Agarwal R, et al. Cardiovascular events with finerenone in kidney disease and type 2 diabetes. N Engl J Med. 2021;385(24):2252-2263. doi:10.1056/NEJMoa2110956 34449181

[22] Bakris GL , Agarwal R, Anker SD, et al. Effect of finerenone on chronic kidney disease outcomes in type 2 diabetes. N Engl J Med. 2020;383(23):2219-2229. doi:10.1056/NEJMoa2025845 33264825

[23] Grune J , Beyhoff N, Smeir E, et al. Selective mineralocorticoid receptor cofactor modulation as molecular basis for finerenone’s antifibrotic activity. Hypertension. 2018;71(4):599-608. doi:10.1161/HYPERTENSIONAHA.117.10360 29437893

[24] Barrera-Chimal J , Estrela GR, Lechner SM, et al. The myeloid mineralocorticoid receptor controls inflammatory and fibrotic responses after renal injury via macrophage interleukin-4 receptor signaling. Kidney Int. 2018;93(6):1344-1355. doi:10.1016/j.kint.2017.12.016 29548765

[25] Kolkhof P , Delbeck M, Kretschmer A, et al. Finerenone, a novel selective nonsteroidal mineralocorticoid receptor antagonist protects from rat cardiorenal injury. J Cardiovasc Pharmacol. 2014;64(1):69-78. doi:10.1097/FJC.0000000000000091 24621652

[26] Kolkhof P , Joseph A, Kintscher U. Nonsteroidal mineralocorticoid receptor antagonism for cardiovascular and renal disorders − new perspectives for combination therapy. Pharmacol Res. 2021;172:105859. doi:10.1016/j.phrs.2021.105859 34461222

[27] Agarwal R , Kolkhof P, Bakris G, et al. Steroidal and non-steroidal mineralocorticoid receptor antagonists in cardiorenal medicine. Eur Heart J. 2021;42(2):152-161. doi:10.1093/eurheartj/ehaa736 33099609 PMC7813624

[28] Pitt B , Kober L, Ponikowski P, et al. Safety and tolerability of the novel non-steroidal mineralocorticoid receptor antagonist BAY 94-8862 in patients with chronic heart failure and mild or moderate chronic kidney disease: a randomized, double-blind trial. Eur Heart J. 2013;34(31):2453-2463. doi:10.1093/eurheartj/eht187 23713082 PMC3743070

[29] Filippatos G , Anker SD, Bohm M, et al. A randomized controlled study of finerenone vs. eplerenone in patients with worsening chronic heart failure and diabetes mellitus and/or chronic kidney disease. Eur Heart J. 2016;37(27):2105-2114. doi:10.1093/eurheartj/ehw132 27130705 PMC4946749

[30] Bakris GL , Agarwal R, Chan JC, et al. Effect of finerenone on albuminuria in patients with diabetic nephropathy: a randomized clinical trial. JAMA. 2015;314(9):884-894. doi:10.1001/jama.2015.10081 26325557

[31] Agarwal R , Filippatos G, Pitt B, et al. Cardiovascular and kidney outcomes with finerenone in patients with type 2 diabetes and chronic kidney disease: the FIDELITY pooled analysis. Eur Heart J. 2022;43(6):474-484. doi:10.1093/eurheartj/ehab777 35023547 PMC8830527

[32] Rossing P , Filippatos G, Agarwal R, et al. Finerenone in predominantly advanced CKD and type 2 diabetes with or without sodium-glucose cotransporter-2 inhibitor therapy. Kidney Int Rep. 2022;7(1):36-45. doi:10.1016/j.ekir.2021.10.008 35005312 PMC8720648

[33] Bayer AG. A study to learn how well the treatment combination of finerenone and empagliflozin works and how safe it is compared to each treatment alone in adult participants with long-term kidney disease (chronic kidney disease) and type 2 diabetes (CONFIDENCE). ClinicalTrials.gov. Accessed December 1, 2022. https://classic.clinicaltrials.gov/ct2/show/NCT05254002

[34] Green JB , Mottl AK, Bakris G, et al. Design of the COmbinatioN effect of FInerenone anD EmpaglifloziN in participants with chronic kidney disease and type 2 diabetes using a UACR Endpoint study (CONFIDENCE). Nephrol Dial Transplant. 2023;38(4):894-903. doi:10.1093/ndt/gfac198 35700142 PMC10064838

[35] Rossing P , Agarwal R, Anker SD, et al. Efficacy and safety of finerenone in patients with chronic kidney disease and type 2 diabetes by GLP-1RA treatment: a subgroup analysis from the FIDELIO-DKD trial. Diabetes Obes Metab. 2022;24(1):125-134. doi:10.1111/dom.14558 34580995 PMC9293162

[36] Heinig R , Gerisch M, Engelen A, Nagelschmitz J, Loewen S. Pharmacokinetics of the novel, selective, non-steroidal mineralocorticoid receptor antagonist finerenone in healthy volunteers: results from an absolute bioavailability study and drug-drug interaction studies in vitro and in vivo. Eur J Drug Metab Pharmacokinet. 2018;43(6):715-727. doi:10.1007/s13318-018-0483-9 29779093

[37] Lentini S , Heinig R, Kimmeskamp-Kirschbaum N, Wensing G. Pharmacokinetics, safety and tolerability of the novel, selective mineralocorticoid receptor antagonist finerenone – results from first-in-man and relative bioavailability studies. Fundam Clin Pharmacol. 2016;30(2):172-184. doi:10.1111/fcp.12170 26604072

[38] Heinig R , Lambelet M, Nagelschmitz J, Alatrach A, Halabi A. Pharmacokinetics of the novel nonsteroidal mineralocorticoid receptor antagonist finerenone (BAY 94-8862) in individuals with mild or moderate hepatic impairment. Eur J Drug Metab Pharmacokinet. 2019;44(5):619-628. doi:10.1007/s13318-019-00547-x 30825073

[39] Palmer BF , Carrero JJ, Clegg DJ, et al. Clinical management of hyperkalemia. Mayo Clin Proc. 2021;96(3):744-762. doi:10.1016/j.mayocp.2020.06.014 33160639

[40] Kardalas E , Paschou SA, Anagnostis P, Muscogiuri G, Siasos G, Vryonidou A. Hypokalemia: a clinical update. Endocr Connect. 2018;7(4):R135-R146. doi:10.1530/EC-18-0109 29540487 PMC5881435

[41] Kovesdy CP , Appel LJ, Grams ME, et al. Potassium homeostasis in health and disease: a scientific workshop cosponsored by the National Kidney Foundation and the American Society of Hypertension. Am J Kidney Dis. 2017;70(6):844-858. doi:10.1053/j.ajkd.2017.09.003 29029808

[42] Ouwerkerk W , Voors AA, Anker SD, et al. Determinants and clinical outcome of uptitration of ACE-inhibitors and beta-blockers in patients with heart failure: a prospective European study. Eur Heart J. 2017;38(24):1883-1890. doi:10.1093/eurheartj/ehx026 28329163

[43] Natale P , Palmer SC, Ruospo M, Saglimbene VM, Strippoli GF. Potassium binders for chronic hyperkalaemia in people with chronic kidney disease. Cochrane Database Syst Rev. 2020;6(6):CD013165. doi:10.1002/14651858.CD013165.pub2 32588430 PMC7386867

[44] Hunter RW , Bailey MA. Hyperkalemia: pathophysiology, risk factors and consequences. Nephrol Dial Transplant. 2019;34(suppl 3):iii2-iii11. doi:10.1093/ndt/gfz206 31800080 PMC6892421

[45] Baker M , Perazella MA. NSAIDs in CKD: are they safe? Am J Kidney Dis. 2020;76(4):546-557. doi:10.1053/j.ajkd.2020.03.023 32479922

[46] Blankenburg M , Pelkey R, Folse HJ. Patient benefits and cost savings predicted for mineralocorticoid-receptor antagonist treatment of early and advanced diabetic kidney disease. Value Health. 2015;18(7):PA508. doi:10.1016/j.jval.2015.09.1458

[47] Pochopień MT , Cherney DZI, Folkerts K, et al. FINE-CKD model to evaluate economic value of finerenone in patients with chronic kidney disease and type 2 diabetes. Am J Manag Care. 2021;27(20 suppl):S375-S382. doi:10.37765/ajmc.2021.88808 34878755

